# Biobehavioral and psychosocial stress changes during three 8–12 month spaceflight analog missions with Mars-like conditions of isolation and confinement

**DOI:** 10.3389/fphys.2022.898841

**Published:** 2022-12-07

**Authors:** Jocelyn Dunn Rosenberg, Amber Jannasch, Kim Binsted, Steven Landry

**Affiliations:** ^1^ School of Industrial Engineering, Purdue University, West Lafayette, IN, United States; ^2^ Metabolite Profiling Facility, Bindley Bioscience Center, Purdue University, West Lafayette, IN, United States; ^3^ Information and Computer Sciences Department, University of Hawaii at Manoa, Honolulu, HI, United States; ^4^ The Harold and Inge Marcus Department of Industrial and Manufacturing Engineering, The Pennsylvania State University, University Park, PA, United States

**Keywords:** stress, isolation, confinement, dopamine, serotonin, cortisol, activity, sleep

## Abstract

Prior theories about individual and team adaptation to living and working in an isolated and confined environment (ICE) have been derived from the experiences of individuals who winter-over in Antarctica or deploy for long durations in submarines. These theories are typically described as a 3- to 4-stage process with phases of excitement and elevated alertness, then followed by difficult phases, including depression and volatility. To further evaluate the applicability of these theories to long-duration human spaceflight missions, longitudinal stress responses to prolonged isolation and confinement of three 6-person crews during 8–12 months simulated Mars missions were characterized through metabolite profiling (biomarkers in hair and urine samples), wearables monitoring (sleep and activity levels), and self-reported ratings of stress, mood, social participation, and perceived health. These data were normalized, aggregated, and clustered to analyze longitudinal trends in biobehavioral and psychosocial stress measures. As a result, this analysis presents a theoretical model that triangulates aspects of prior theories with new evidence to describe ICE stress at HI-SEAS as 1) eustress of initial adaptation (high stress hormone levels at mission start), 2) deprivation due to prolonged isolation and confinement (decreasing dopamine and serotonin levels), 3) disruption of individual and team dynamics (changes in activity levels, mood, perceived stress, and social participation) and 4) asynchronous coping (changes in sleep-wake cycles, outlook, and team cohesion). These findings support several aspects of prior theories in combination, such as the elevated alertness at mission start and that adverse conditions are most likely to develop after the halfway point of a mission (e.g. for HI-SEAS 8–12 months missions, after approximately 6 months) followed by a period of volatility until the end (e.g. as stated in Rohrer’s theory, ups and downs until the end, not a renewed outlook at the end as described in 3rd quarter phenomenon theory).

## Introduction

Stress responses occur due to environmental stressors (e.g. isolation and confinement) as well as psychological, social, biological, and behavioral patterns. Biobehavioral and psychosocial measures of stress over time were analyzed to further develop and evaluate existing theories on adaptation to isolation and confinement. Theories about adaptation to isolation and confinement are typically described as a 3- to 4-stage process that includes some, or all, of the following reactions: alarm, alertness, novelty, resistance, depression, exhaustion, volatility, and renewed outlook (Fraisse and Rasmussen, 1973). These theories of adaptation to living in isolation and confinement are derived from the experiences of individuals who winter-over in Antarctica or deploy in submarines (Harrison et al., 1991; Decamps and Rosnet, 2005).

One theory for how human psychology adapts to long-duration isolation and confinement is the “3rd quarter phenomenon” (Bechtel and Berning, 1991). This theory states that after the half-way point of an expedition, the novelty of a new mission has faded away, yet there is still a long period of time remaining until the end; thus, the 3rd quarter is characterized as a lull, or a difficult phase psychologically until reaching the 4^th^ quarter with renewed perspective that the end is near, or there’s a “light at the end of the tunnel” (Bechtel and Berning, 1991). Another theory, the “relief from pressure” hypothesis is analogous to a pot of water on a stove that will boil over if the heat source is not turned down; there's a relaxation down of social norms, or in other words, there's a change or leniency that develops to recover from the chronically elevated stress levels. Finally, Rohrer’s theory describes three phases of adaptation, first, elevated alertness, then a sense of loss and regret for putting oneself or loved ones through this difficulty, and finally, volatility, or highs and lows until the end (Altman and Haythorn, 1967).

The applicability of these prior theories to Mars mission context may be limited due to differences in overall mission design and logistics, such as group size (much smaller group for Mars crew compared to Antarctic expeditions and submarine deployments), mission duration (longer duration for Mars mission), vehicle size (more confined living space for Mars mission), communication delays) and other differences listed in Table 1. Furthermore, it is likely that each group and mission (e.g. first Mars missions vs. the 10th, or Lunar vs. Martian) will have idiosyncratic differences based on the mission objectives, concept of operations, crew composition (e.g. various cultural and technical backgrounds and interactions of various personalities), social norms or code of conduct (e.g. daily living habits), and the various accomplishments and failures that occur during the mission, including major life events (such as the sudden loss of a loved one).

**TABLE 1:** Comparing characteristics of Mars-like isolation and confinement (highlighted in orange) with missions to the Moon and ISS, with spacelight analog environments: NEEMO, HERA, MDRS, and HI-SEAS, and with terrestrial submarine deployments and expeditions to Antarctica.

Environment	Number of people	Transit time	Station time	Evacuation time	Habitable volume	Ability to separate?	Communication delay
Mars	4 to 6	9 months	15 months	9 months	size of small RV	No	3–22 min
Moon	4 to 6	3 days	<12 months	3 days	size of small RV	No	1.25 s
ISS	3 to 6	1 day	∼6 months	3 days	size of a house	Somewhat	Intermittent loss of signal
Submarine	50 to 200	30 min	3–6 months	30 min	size of cruise ship	Yes	None
Antarctica	50 to 1200	3 days	4–5 or 13 months	3 days	size of University	Yes	None
HI-SEAS	6	30 min	4–12 months	30 min	size of an apartment	No	20 min
NEEMO	6	30 min	<2 weeks	30 min	size of small RV	No	Sometimes
HERA	4	5 min	<45 days	5 min	size of an apartment	No	Sometimes
MDRS	6	10 min	<2 weeks	10 min	size of an apartment	No	Sometimes

Accumulation of stressors over time and contagion effects of stress on team dynamics are investigated here through biobehavioral and psychosocial monitoring of small teams living and working in Mars-like isolation and confinement. In general, few longitudinal studies have been conducted for quantifying how individuals’ biological metabolites, psychological outlook, social drive, and behavioral habits change over a long duration in Mars-like isolation and confinement (Basner et al., 2014; Nicolas and Gushin, 2015). In collaboration with Hawaii Space Exploration Analog and Simulation (HI-SEAS) missions, this work is analyzing *n* = 18 individuals from three 8–12 months simulated Mars missions, each with six-person, mixed gender, international crews to evaluate the applicability of existing theories about phases of adaptation to isolation and confinement through analysis of stress responses.

HI-SEAS provides a ground analog baseline for human spaceflight, with small group living, delayed communications, shelf stable food, limited entertainment, etc. but without the harmful effects of microgravity and radiation exposure. This provides a baseline for assessing the impact of isolation and confinement alone, without exacerbating factors due to environmental alterations, such as radiation and weightlessness. This research aims for a balanced approach of bottom-up data analysis and top-down theoretical model development based on the findings in these HI-SEAS stress data (Huang, 2018). For the context of stress physiology research, the environment of a Mars analog mission is an intermediate between laboratory work and real-world application. Stress research often takes place in a controlled, laboratory environment where researchers actively introduce a physical, mental, or social stressor and then measure human or animal responses. Rather than artificially-inducing stress in a laboratory, HI-SEAS is an opportunity for rigorous study of how stress naturally develops in a real but limited context. The HI-SEAS environment is not as complex and varied as in the “real world” which has innumerable lists of possible impacts on health and performance, such as meeting people, trying new foods, traffic, toxins, etc. In the isolated and confined environment of HI-SEAS, human behavior is taking place in a more limited context with a static food supply, predictable schedules, isolated with only six people, habitat monitoring, and well-defined modes of physical activity, diet, leisure, and entertainment, enabling a rich data set and documented context for this stress research investigation.

### Background on HI-SEAS

At HI-SEAS, Mars-like isolation and confinement is maintained for a long duration in a secluded area located at a high elevation (8,200 feet) on Mauna Loa volcano where plants and animals are scarcely present. The HI-SEAS habitat is a geodesic dome nestled in between ridges of volcanic terrain that is similar to the basalt of Mars, with a confined living space of 1,300 square feet. The walls are thin and without sound proofing, so the habitat design is lacking space for private time to recharge in solitude, exacerbating the environmental and psychosocial stressor of confinement for crew members (Bassingthwaighte, 2017). Each crewmember has very limited, private quarters (less than 50 square feet, smaller than a walk-in closet). The floor plan also includes a main living area (for work, entertainment, and exercise), a laboratory, 1.5 bathrooms (with composting toilets), a kitchenette (with a microwave, hot plate, and toaster oven), and a small dining area.

While isolated from the rest of the world, crewmembers developed their own social activities, such as sharing meals together, watching movies, dancing, or playing board games, and also collaborated on work projects and participated in exercise programs as a group (or sub-group) (Heinicke et al., 2021). Food, supplies, and medications were delivered to the crew on a resupply schedule (with the drop-off of the supplies executed quietly and at odd hours to prevent any in-person interaction between the crew and test support). Crew were provided with shelf-stable food ingredients, such as freeze dried meats and dehydrated vegetables (since a real Mars mission will require foods that can survive the 3-year journey to Mars and back to Earth).

Living off the grid with sustainable energy systems required adaptation to conserve energy and resources, especially on cloudy days when not producing as much solar energy, then crew would need to limit the use of powered equipment (computers, displays, treadmills, etc.), kitchen appliances, and HVAC systems (Engler et al., 2019). A web-based data dashboard system called “UILA” was made available for the crew to monitor solar array energy production, battery charge status, and hydrogen back-up system status along with weather station data and water tank levels. Crew typically limited themselves to 6–8 min of total shower time per person each week (so three 2-min showers, two 3-min showers, one 6-min shower, or other combinations); however, when a water resupply was scheduled to occur and spare capacity was still available, then the crew would use up as much water as available in the days leading up to the resupply (Heinicke et al., 2021).

In Table 1, HI-SEAS mission characteristics are compared with other spaceflight analog environments, design reference missions for Mars, Moon, and ISS missions, as well as terrestrial submarine deployments and Antarctic expeditions. It is the only environment that adheres to Mars-like delayed communications. At HI-SEAS, all communication with crew was automatically delayed by an intermediate server to cause messages to lag 20-min each way to mimic the long distance between Earth and Mars. Rather than consistently having a 20-min delay, on a real Mars mission there will be a range from 5–35 min of delay in communication and transmission of data packets (depending on orbital timing as the distance between Earth and Mars varies from 35 million to 250 million miles). The crew acted autonomously with “mission support” teams available 24–7 virtually (but over the 20-min communication delay) to help with providing any test instructions, recordings of news articles, recipes, or other entertainment (sporting events, movies, etc.), and information to address contingencies, such as habitat maintenance and networking updates.

## Materials and methods

In brief, HI-SEAS immerses 6-person crews of astronaut-like individuals in a Mars-like habitat on Mauna Loa volcano, isolated from the rest of humanity for a long duration, confined to a 1,300-square-foot living space, except for spacewalks wearing mock spacesuits, while living on limited energy, water, and food resources, and relying on delayed communications with “Earth” and mission support. HI-SEAS is a NASA-funded study characterizing the behavioral health and performance (BHP) risks for long-duration human spaceflight missions. This stress research is an opportunistic research project that complemented the BHP study (Caldwell, 2016) and was led by researchers at Purdue University (Dunn, 2017a). It was conducted in accordance with the NASA Institutional Review Board (IRB) approved protocol for participant privacy protection. Protocols of this research have been reviewed by the Institutional Review Boards of the University of Hawaii, NASA Johnson Space Center, and Purdue University. The author was added to University of Hawaii and NASA IRB protocols as a key contributor, Principal Investigator of stress research at HI-SEAS. These stress research protocols were also submitted to Purdue University IRB.

Data collection for this stress research at HI-SEAS along with the number of subjects for each data collection type are listed in Table 2. In summary, questionnaires for self reporting on perceived stress were administered for all three missions to compare with stress hormone levels in hair samples (for all three missions). Additionally, urine samples (for two of the missions) were analyzed for metabolites of stress hormones, oxidative damage, and neurotransmitters. For HM5, only hair samples and self-reporting questionnaire data were collected, no urine samples nor wearables data. Wearables data about sleep and activity levels are presented for HM3 and HM4 missions, and this included resting heart rate data for HM4 only.

**TABLE 2:** Summary of data collection and subject participation in the three HI-SEAS missions (HM3, HM4, and HM5).

Data sources	HM3	HM4	HM5	Total subjects
Questionnaires	*n* = 6	*n* = 6	*n* = 6	18
Hair Samples	*n* = 6	*n* = 6	*n* = 6	18
Urine Samples	*n* = 6	*n* = 6		12
Sleep and Actvity	*n* = 6	*n* = 4		10
Resting Heart Rate		*n* = 4		4
Social Participation	*n* = 6			6

This longitudinal, multivariate data was aggregated into “psychosocial” and “biobehavioral” stress measures, as an approach to separating out measurable components of Engel’s biopsychosocial model (Farre and Rapley, 2017). While Engel’s model depicts inner layers starting from genetic composition, biological structure and function, and behavioral determinants, all the way up to outer layers of relationships, community, society, and the environment. This work is analyzing biomarkers, sleep and exercise behaviors, psychological outlook, and social status and presents a suite of protocols, data integration, and measures to bridge the gap between the layers in Engel’s model.

From a biological perspective, the “fight or flight” stress response is a catabolic, energy-releasing process that converts stored glycogen into glucose; for every epinephrine molecule that binds a G-protein-linked cell receptor, it is estimated that 100 G-proteins are activated to initiate phosphorylation chain reactions leading to over 10 million glucose-6-phosphate molecules from each epinephrine molecule (Sherwin and Saccà, 1984; Krantz, 2008). These compounds act on the cells to create a jolt of energy to the system that can enable high performance in the short-term.

Biological markers such as stress hormone metabolites quantify the trajectory over time from acute eustress to chronic distress, but these alone do not fully describe behavioral, social, and psychological patterns underlying stress responses. To fully inform and map the process of how stress initiates, dissipates, or becomes harmful in the long-term, additional measures are needed beyond biological, such as sleep and exercise behaviors, psychological outlook, and social participation.

To contribute evidence toward the need for longitudinal analysis of health and stress states in isolation and confinement, this research presents data from 18 subjects who participated in three HI-SEAS missions: 8-month HI-SEAS mission 3 (HM3), the 12-month HI-SEAS mission 4 (HM4), and the 8-month HI-SEAS mission 5 (HM5) in the 2014–2017 timeframe. The methods are outlined in the following sections on self-reporting questionnaires, wearable wristbands (sleep, activity, and heart rate monitoring), biological samples (hair and urine samples), and data integration approach.

### Self-reporting questionnaires

This stress research protocol administered at HI-SEAS included Qualtrics platform web link to a self-reporting questionnaire/survey with four parts: General Health Questionnaire (GHQ), Perceived Stress Scale (PSS), Ranking how aspects of your life are linked to stress (internal vs. external stressors) (Dunn, 2017a), and Stress Intensity, Frequency, Onset, Recovery (SIFOR) (Dunn, 2021). Data analysis is leveraging prior work on developing metrics for self-reported perceptions of health and stress levels over time (Dunn, 2017a; Dunn, 2021). Social participation was self-reported in questionnaires and logged in operational records through workout participation and social activities schedules created and maintained by crewmembers during the mission (Heinicke et al., 2021).

### Wearables

During HM3 and HM4, crewmembers were equipped with Jawbone wristbands. However, due to hardware failure issues, during the long 12-month HM4, crewmembers were resupplied with new wearables and then switched from Jawbone to Fitbit devices. This presented the need for a study of Jawbone and Fitbit data output differences (Shah, 2017). HM5 did not include any wearables data collection for this stress research. Data analysis is leveraging prior work on defining and computing sleep and activity metrics from wearables data (Dunn, 2017a; Dunn, 2017b; Shah, 2017).

### Biological samples

Hair and urine samples were collected to quantify biomarkers throughout the mission. Crewmembers were instructed to provide first-of-the-morning urine samples on a bi-monthly basis and hair samples on a monthly basis. Hair samples were stored in aluminum foil at room temperature. First-of-the-morning urine samples were collected, then pipetted into 3-ml tubes and stored in the habitat’s standard freezer. At regular resupply intervals, the urine was collected by HI-SEAS staff and shipped on dry ice to Bindley Bioscience Center where they were stored in -80C freezer. For outlier detection, only one data point of a hair sample in one crewmember’s timepoint was determined that it was beyond what is possible range with physiological constraints, and it was removed from analysis, as error was likely caused from a missed step in hair sample preparation. Trends are being analyzed in the aggregate from multiple crewmembers who also had different participation in various types of data collection, such as not providing all samples. However, this data integration approach (*Data Integration Section*) enables normalization, classification, and aggregation of stress indicators from multiple data sources, crew members, and mission durations.

#### Targeted analysis of steroids in hair

Human hair samples were treated and extracted similar to (Gao et al., 2013). At the time of analysis, each hair sample was briefly washed with an excess volume of methanol or isopropyl alcohol to remove oils on the outside of the hair and allowed to dry overnight at room temperature. The samples were then weighed, cut into approximately 5 mm pieces, then transferred to a 2 ml stainless steel bead homogenizer tube for grinding. After grinding each hair sample into a fine powder, 1 ml of methanol was added as a steroid extraction solvent. To each sample 10 µL of an internal standard mixture containing 10 ng of isotopically labeled steroid (^13^C_3_-testosterone and d_4_-cortisol solution in methanol) was added to each sample and vortexed for one minute. The samples were allowed to extract overnight at 4°C. The following day, the samples were vortexed for 10 min and centrifuged at 13,000 g for 10 min. The top organic layer was collected and transferred to a new tube for drying. The samples were dried in a rotary evaporation device at 45°C for 3 h. Each sample was then derivatized with 50 µL of Amplifex keto reagent (# 4465962, AB Sciex, Framingham, MA) according to the kit directions just prior to instrument analysis. The internal standards ^13^C_3_-testosterone (# T-070) and d_4_-cortisol (#C696302) were purchased from Sigma Aldrich (St.Louis, MO) and Toronto Research Chemicals (Ontario, Canada) respectively.

An Agilent 1260 Rapid Resolution liquid chromatography (LC) system coupled to an Agilent 6460 series QQQ mass spectrometer (MS/MS) was used to analyze the steroids in each hair sample extract (Agilent Technologies, Santa Clara, CA). An Agilent Eclipse plus C18 2.1 mm × 50 mm, 1.8 µm column was used for LC separation. The buffers were (A) water +0.1% formic acid and (B) acetonitrile +0.1% formic acid. The linear LC gradient was as follows: time 0 min, 10% B; time 1.0 min, 10% B; time 11 min, 100% B; time 12 min, 100% B; time 13 min, 10% B; time 15 min, 10% B. The flow rate was 0.3 ml/min. Multiple reaction monitoring was used for MS analysis. The data were acquired in positive electrospray ionization (ESI) mode for targeting compounds based on precursor and product ion (m/z), collision energy, and polarity. The jet stream ESI interface had a gas temperature of 325°C, gas flow rate of 8 L/min, nebulizer pressure of 45 psi, sheath gas temperature of 250°C, sheath gas flow rate of 7 L/min, capillary voltage of 4000 V in positive mode, and nozzle voltage of 1000 V. The ΔEMV voltage was 500 V. Agilent Masshunter Quantitative analysis software was used for data analysis (version 8.0).

#### Targeted analysis of neurotransmitters, stress hormones, and oxidative damage in urine

The urine samples were preserved at pH < 6 by adding hydrochloric acid (HCl) for long term storage in the freezer. Internal standards (N-acetyl-S-3-hydroxypropylcysteine (3HPMA)-d3, 5-hydroxyindoleacetic acid (5-HIAA)-d5, 6-Sulfatoxymelatonin (6SOM)-d4, dopamine-d4, metanephrine-d3, normetanephrine-d3, serotonin-d4, vanillylmandelic acid (VMA)-d3) were purchased from Sigma Aldrich, St. Louis, MO. Stock solutions were prepared in methanol and stored at -80°C when not in use. Samples were prepared with a simple dilute and shoot protocol similar to (Shen et al., 2015). Each urine sample was spiked with 100 ng of internal standard mixture, then mixed 1:1 (v/v) with water, and finally centrifuged at 13,000 g for 10 min just prior to analysis on the LC/MS/MS instrument.

An Agilent 1260 Rapid Resolution liquid chromatography (LC) system coupled to an Agilent 6470 series QQQ mass spectrometer (MS/MS) was used to analyze the neurotransmitters. (Agilent Technologies, Santa Clara, CA). A Waters T3 2.1 mm × 150 mm, 3.0 µm column was used for LC separation (Water Corp, Milford, MA). The buffers were A) water +0.1% formic acid and B) acetonitrile +0.1% formic acid. The linear LC gradient was as follows: time 0 min, 0% B; time 3 min, 0% B; time 10 min, 11% B; time 14 min, 70% B; time 15 min, 0% B; time 20 min, 0% B. The flow rate was 0.3 ml/min. Multiple reaction monitoring was used for MS analysis. Data were acquired in positive/negative electrospray ionization (ESI) modes. The jet stream ESI interface had a gas temperature of 325°C, gas flow rate of 8 L/min, nebulizer pressure of 40 psi, sheath gas temperature of 250°C, sheath gas flow rate of 7 L/min, capillary voltage of 4000 V in positive mode, and nozzle voltage of 1500 V, capillary voltage of 3000 V in negative mode, and nozzle voltage of 500 V. The ΔEMV voltage was 400 V. Agilent Masshunter Quantitative analysis software was used for data analysis (version 8.0).

### Data integration

Data were combined to optimize integration for multi-variate dataset which including some missing data points (e.g. no urine samples for HM5, some urine samples are missing for HM4 due to shipping errors and wearables data are missing for some time periods due to device malfunctions or participant errors). To overcome the experimental errors and limitations, data from multiple stress indicators are aggregated to capture the likelihood of stress for a given timepoint in these HI-SEAS missions based on all data available, which are normalized before combining to overall percentage of stress indicators. This percentage of indicators that are in 1: “stressed” state vs. 0: “not stressed” state represents the likelihood of stress for the crew at that point in time. Given that existing theories have focused on 3–4 phase models over long durations, data were analyzed at the level of weekly (if daily or weekly data available) or monthly time points (if only have data points biweekly or monthly data available) in order to enable aggregation and comparison of the phases of the mission (e.g. thirds or quarters). To ensure equal weighting of each type of data, this aggregation first was performed at the level of each data stream to the final timescale, e.g. daily heart rate 1 vs. 0 states were averaged for each week then compared with weekly stress survey results.

**FIGURE 1:**
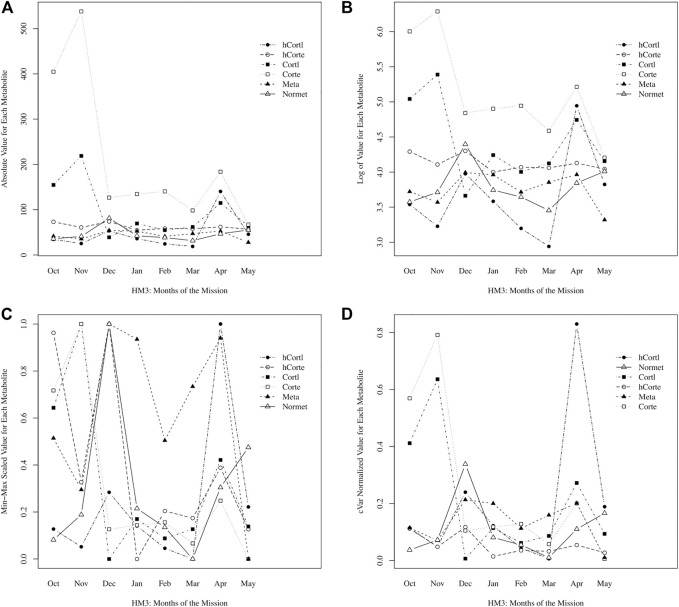
Comparing data normalization methods: **(A)** raw absolute values of data, **(B)** log normalized, **(C)** min-max scaling, **(D)** cVar normalization.

Here, a novel approach to data normalization (called “cVar normalization”) is presented. It is a modified min-max scaling with the coefficient of variation (cVar) as a weighting factor for each data stream. As an example for Participant A, stress hormones and metabolites were plotted raw, with a log-transformation, with standard normalization, and with cVar- normalization in Figure 1. With the intended goal of peak detection, or identifying timepoints with significantly higher stress values, it is immediately clear that the min-max scaling approach is non-specific, creating significant peaks in every data stream. Log-transformation performed a scaling of the data that enables comparison; however, the cVar-normalization found specific peaks of interest that log-transformation did not, namely hair cortisol levels in April. In cross-referencing self-reporting questionnaire data, April was perceived as the most stressful month of the mission for Participant A; therefore, this supports that cVar-normalization identified a valid increase to a peak value in stress.

Normalization enables data integration for multivariate, longitudinal data streams of varying magnitude and distribution. As shown in Eq. 1, min-max scaling adjusts data to a range from 0 to 1, but then data are being scaled to 95% of the minimum, such that normalized data will approach zero but not equal zero (to avoid errors if normalized data streams are divided). After data are normalized to a range that is nearly 0 to 1, then each data stream is weighted by its coefficient of variation (cVar). Recall that cVar is the standard deviation divided by the average of the data stream. With the intended goal of identifying time points with significantly higher stress values, this approach is normalizing the magnitude of the data while also weighting by cVar to appropriately scale the variation in each data stream. For example, if a data stream has low variation of less than 1% difference between min and max value, then it is a misrepresentation to have these data normalized from nearly 0 to 1 alongside values that had 50% difference between min and max value (but also get represented as a spread between nearly 0 to 1); thus, cVar weighting enables proportionate representation of variation, such that values need not span the entire 0 to 1 range if there is low variation, and conversely, values can extend beyond 1 for the case of extreme variations.

$$D_{norm}=\frac{stdev\left(D\right)}{avg\left(D\right)}\left(\frac{D-0.95*\min\left(D\right)}{\max\left(D\right)-0.95*\min\left(D\right)}\right)$$

Identifying and labeling as stress indicators is achieved by k-means clustering for maximizing the difference between the mean values in the group of data that is classified as “stress indicator” for each individual and data stream. Using Matlab k-means function, clustering was performed to identify stress indicators in each of the normalized data streams to identify and label stress indicators at various measured time points of *D(t,p,m)* for each participant *p* and measure *m*. First each data stream for each participant was grouped into 2 or 3 clusters, depending on total number of data points, specifically for daily or weekly datasets then enough n for 3 clusters (and only 2 clusters for monthly datasets). In the case of 2 clusters, then one group was the “high value” and the other the “low value,” but for the case of 3 clusters, then there’s an additional “mid value” group. To map to the final labeling of data with binary stress indicator (1: stressed, 0: not stressed), subject matter expertise about the data stream, such as how the behavior (e.g. sleep) or metabolite (e.g. normetanephrine) is up or down regulated during physiological stress responses, is required to determine if high values or low values indicate stress, as shown in Table 3.

**TABLE 3:** Biobehavioral and psychosocial stress indicators (binary measures).

**Binary measures**	**Biobehavioral stress**	**Psychosocial stress**	In bold if higher value indicates stress, and italicized if low value indicates stress
Stress Biomarkers	X		**Cortisol, Cortisone, Metanephrine, Normetanephrine, Vanillylmandelic Acid (VMA)**
Oxidative Damage	X		**3-hydroxypropylmercapturic acid (3-HPMA), Malondialdehyde (MDA)**
Circadian Dysruption	X		*Sleep duration, Sleep quality, 6-sulfatoxymelatonin* (*6-SOM*)
Mood Changes		X	*Serotonin, Dopamine, Testosterone*
Perceived Health		X	*General Health Questionnaire (GHQ)*
Perceived Stress		X	**Perceived Stress Scale (PSS), Stress Intensity Frequency Onset Reovery (SIFOR)**
Physical Activity	X		*Steps, Activity Score*
Resting Heart Rate	X		**First-of-morning Resting Heart Rate (RHR)**
Social Participation		X	*Group Social Activities*

**FIGURE 2:**
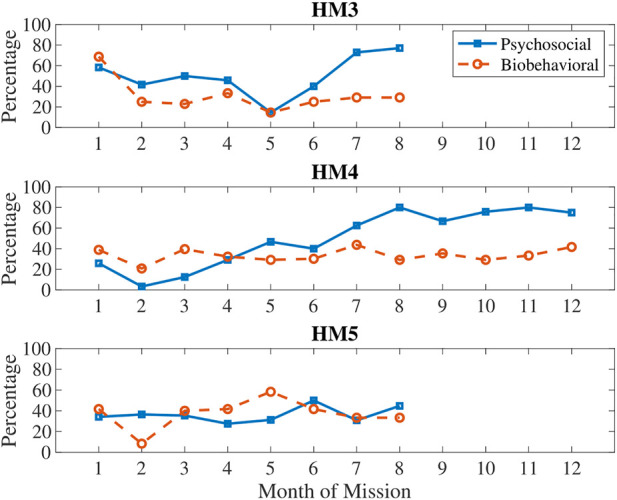
Comparing biobehavioral and psychosocial stress indicators for HM3, HM4, and, HM5.

**FIGURE 3:**
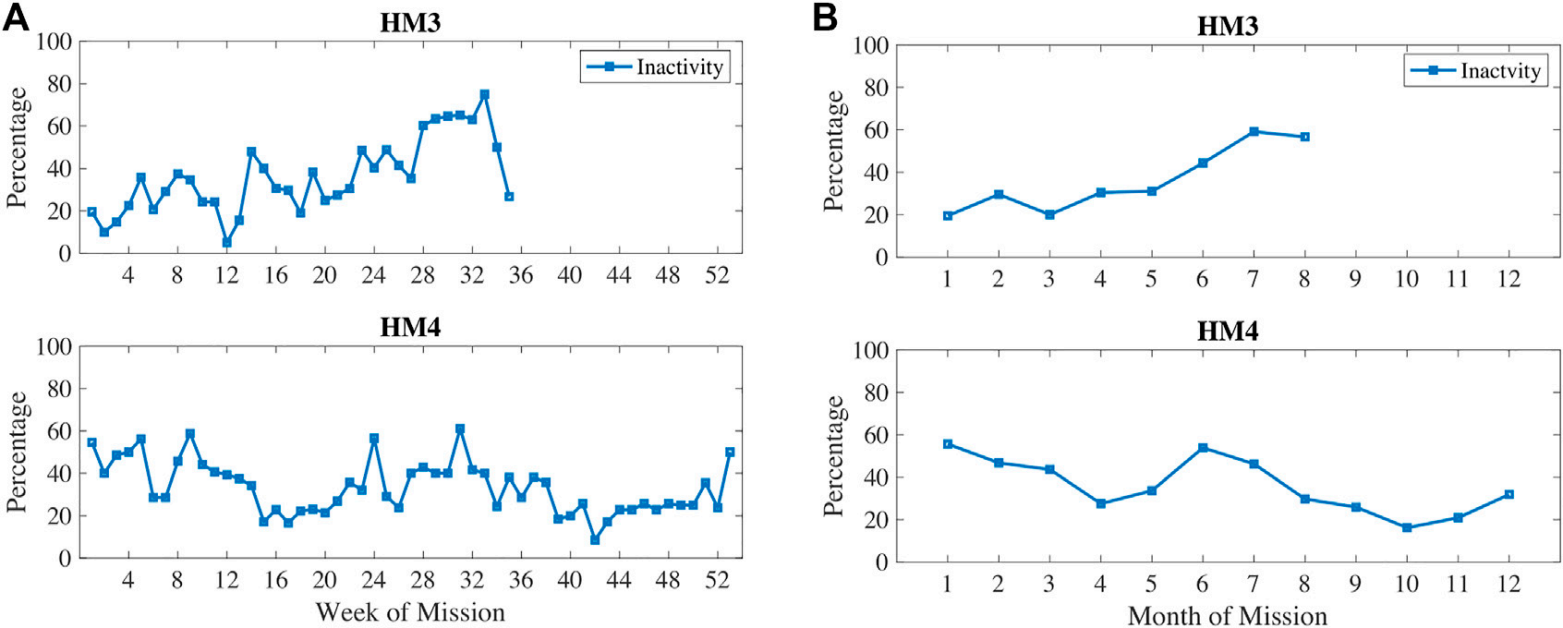
Percentage of measures classified as low physical activity measured *via* wearable devices during HM3 and HM4 and aggregated here on **(A)** weekly and **(B)** monthly basis.

**FIGURE 4:**
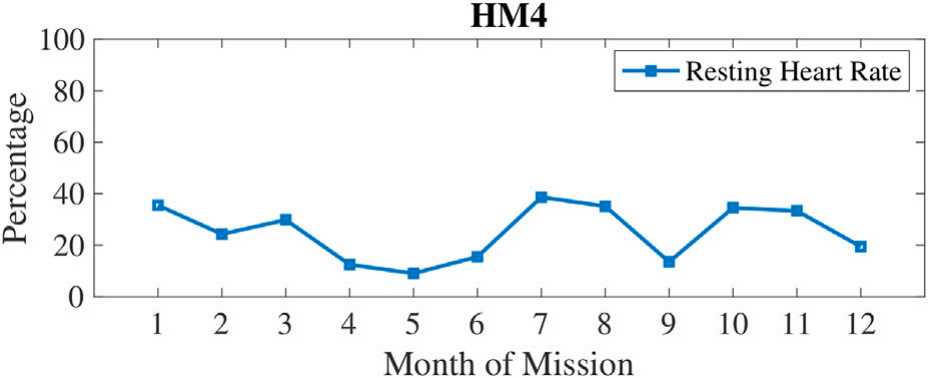
Percentage of measures classified as high Resting Heart Rate (RHR) which was measured by wrist-worn wearables automatically first of the morning upon detection of awakening.

**FIGURE 5:**
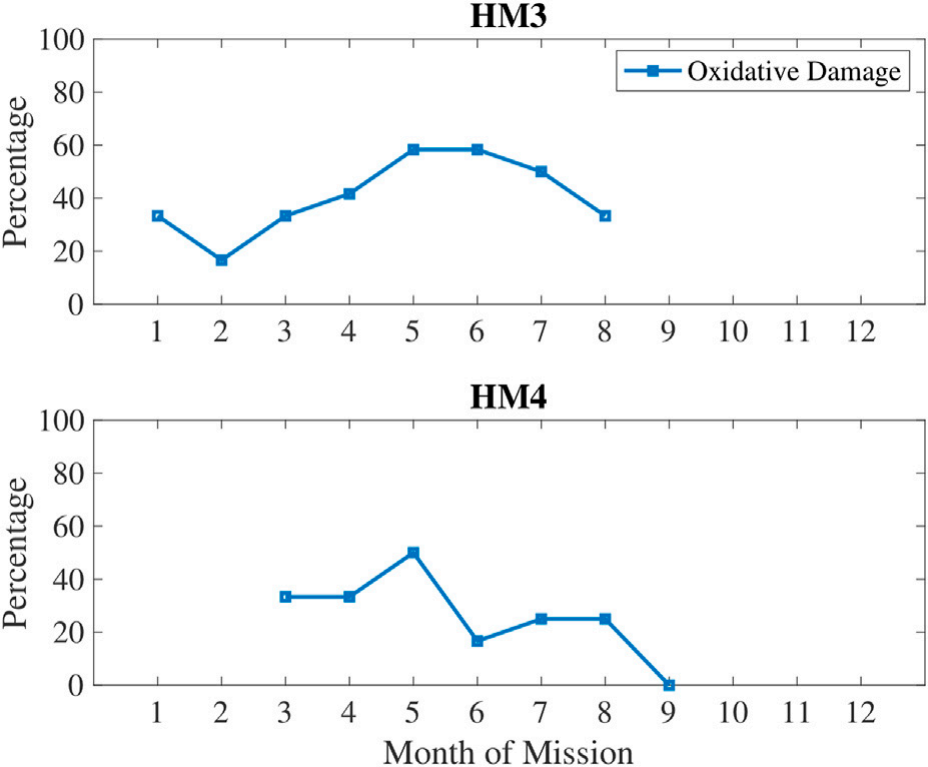
Percentage of measures classified as high oxidative damage quantified in urine *via* 3-HPMA biomarker.

**FIGURE 6:**
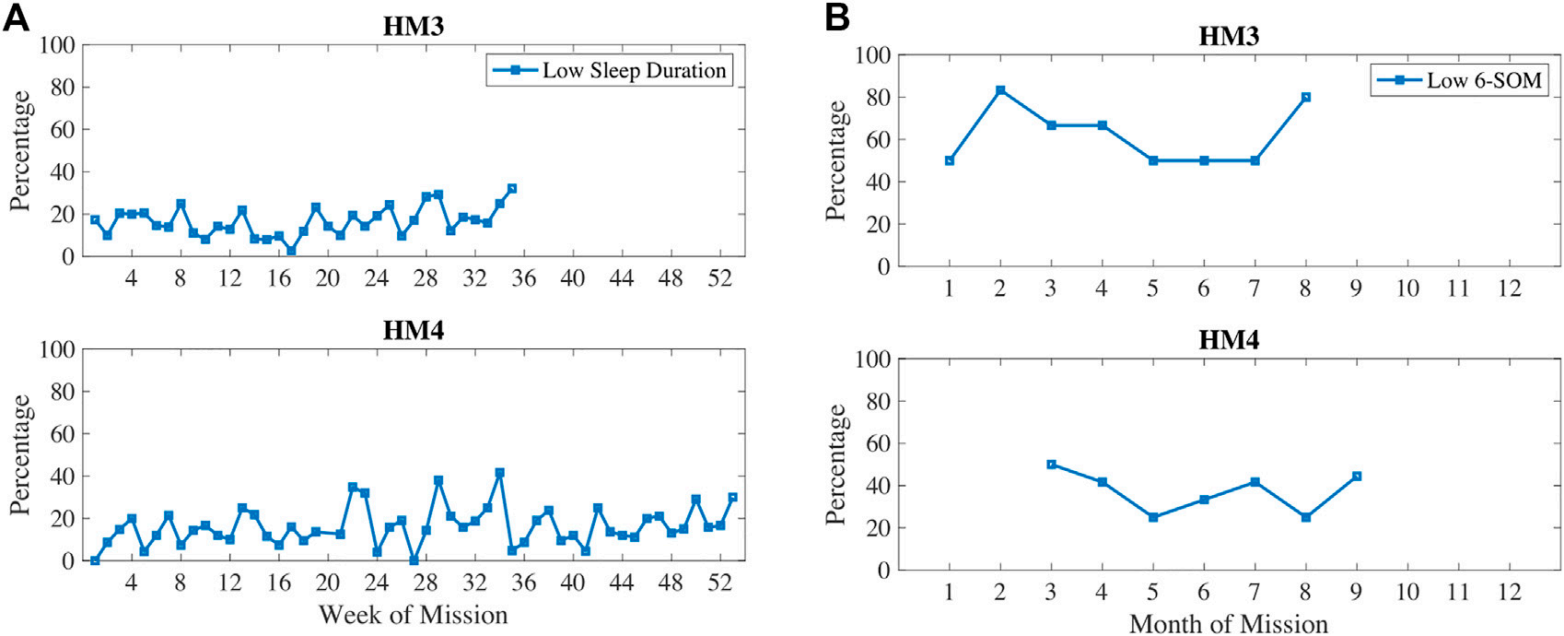
Percentage of measures classified as low sleep duration and/or low circadian biomarker 6-sulfatoxymelatonin (6-SOM).

**FIGURE 7:**
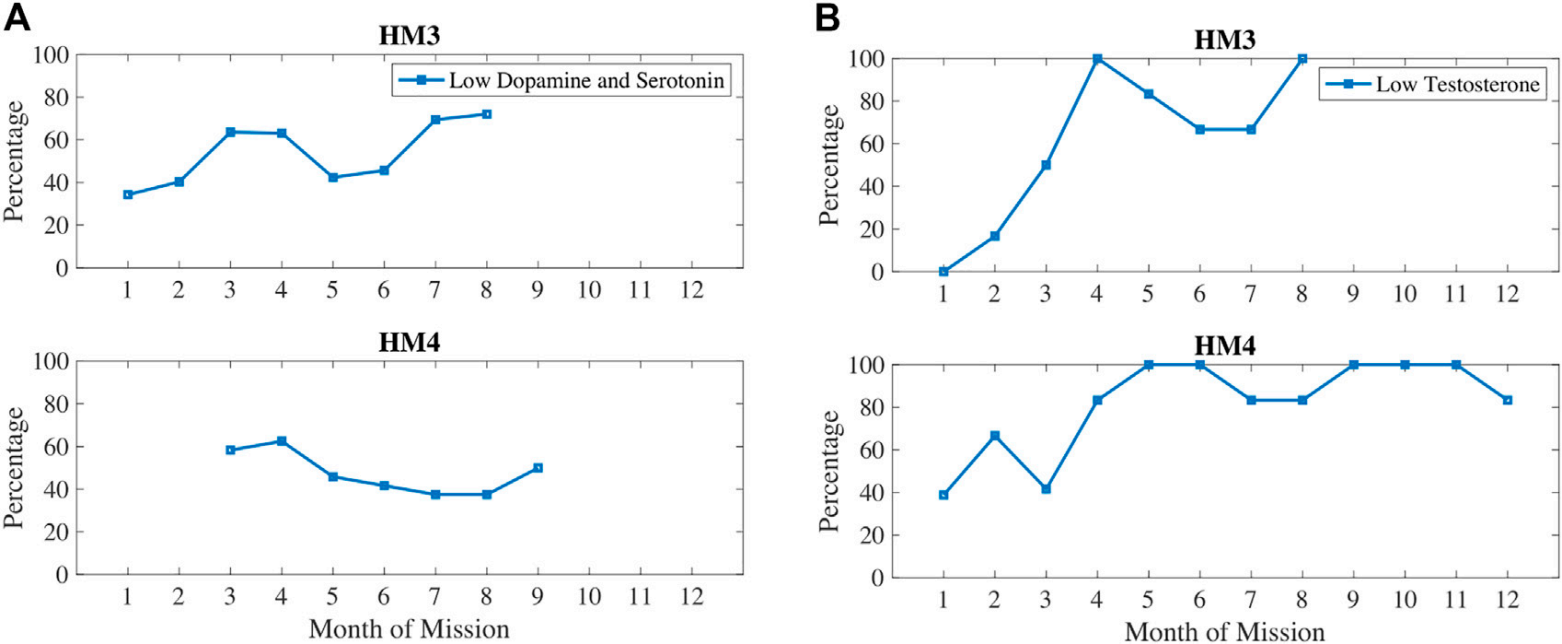
Percentage of measures classified as low dopamine and serotonin **(A)** and/or low testosterone **(B)**.

The plots of results to follow in Figures 2–7 are presented with the *y*-axis being “percentage of stress indicators” (or percentage of data points classified as 1: stress) among all of the data available for each timepoint (or the likelihood of being stressed over time). For example, rather than “sleep duration” on the *y*-axis, the stress indicator of “low sleep duration” is presented such that the higher the percentage on each of these plots, the stronger the indication of stress. If all of the crewmembers and all of the measures for a given timepoint were classified as stress, then it will show 100% whereas if only half of the crewmembers at a given time point for a plot with only one measure, such as testosterone, are classified as stress (or conversely, if half of the measures for all of the crewmembers) then it will show 50%. This enables aggregation of all participant data and comparison of missions that have varying measures (as shown in Table 2).

### Theoretical models

For comparing existing theories of adaptation to isolation and confinement with HI-SEAS data, these models were translated into stress trajectories. The theories are described as 3-4 stages with various psychological responses during each stage. These psychological states were mapped to stress, such as mapping a period of depression and regret as stress and a period of relaxation as not stress. The models were first created to match the trends of the 3-4 stages described through mapping of the constructs in each theory to stress indicators defined in Table 3.

Then each of the theories mapped into a piecewise function that was shaped to match the theoretical description (e.g. high stress in 3rd quarter of the mission for the 3rd quarter phenomenon theory). These piecewise functions were scaled to have the same total area under the curve for each theoretical model in order to describe the various timing or stages of the *same total stress* of a given mission. These data sources presented next in the Results section map these shapes to a piecewise function of the trends, the HI-SEAS theoretical model was also scaled to have the same area under the curve as the other theoretical models. The HI-SEAS “combined stress” model is shown alongside a combined “average” that is equally weighting all of the models.

## Results

Here, indicators of stress are derived from a range of data sources, including questionnaires, biological samples, and wearables. This work has identified stress events over time by analyzing indicators of the construct of stress, such as high stress hormone levels or low activity levels. In summary, stress indicators are derived from analyzing the following: stress hormones and metabolites, sleep and activity, oxidative status, resting heart rate, social participation, mood and neurotransmitter levels, and self-reported perceived stress and health (as listed in Table 3).

In Figure 2, biobehavioral and psychosocial measures are compared on the same plot to show differences in these types of stress (see Table 3 for categorization of measures by type of stress). Generally, biobehavioral stress was highest in the first month and around the halfway point of the mission, whereas psychosocial stress accumulated over time with sharp decreases just before the halfway point and then sharp increases after the halfway point of the mission.

In Figure 3, physical inactivity (or low physical activity) is plotted with weekly 1) and monthly 2) aggregation. Inactivity was most prevalent at 6–7 months into these missions. In Figure 4, resting heart rate (RHR) data is aggregated and follows a similar trend with higher RHR during the 6–7 months time period of inactivity. Note that the wearable devices were replaced with another model for the last 3 months of HM4 mission, and therefore, the activity and RHR for months 10–12 is not directly comparable to the first 9 months (Shah et al., 2017).

In Figure 5, oxidative damage biomarkers are shown to increase up until the 5–6 months time period then decreased. This aligns with the inactivity and RHR data. In Figure 6, low sleep and circadian measures are plotted, namely weekly sleep duration and a metabolite of melatonin, 6-sulfatoxymelatonin (6-SOM) which is also an antioxidant. Sleep durations were cyclical on a monthly basis. 6-SOM was increasing during the 3–5 months timeframe and lower during low sleep time periods, such as during the second month and the last month of HM3. Oxidative damage was lower when 6-SOM was increasing during the 3–5 months timeframe.

In Figure 7, dopamine and serotonin are plotted in 1) and compared with testosterone (b). Dopamine and serotonin were decreasing during the first 3–4 months of the mission. Dopamine and serotonin recovered around the halfway point of the mission but decreased again near the end of the mission. Similarly, testosterone decreased during the first half of the mission, recovered after the halfway point, and then decreased again after 7–8 months of the mission. These trends correlate with sleep and circadian stress measures, such as how 6-SOM, dopamine, serotonin, and testosterone are all increasing around the halfway point of the mission.

**FIGURE 8:**
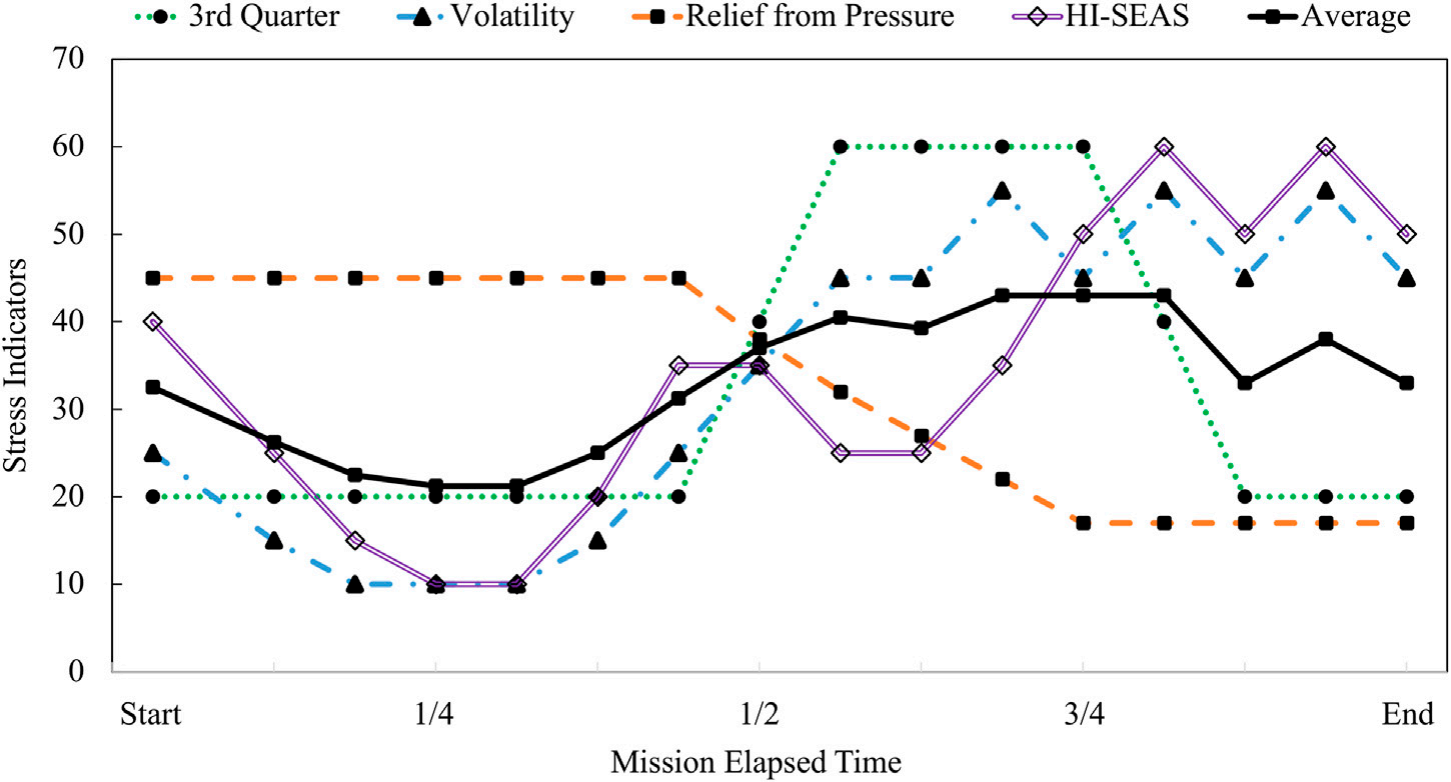
Theoretical models of adaptation to isolation and confinement plotted as stress responses.

In Figure 8, the existing theories of adaptation to isolation and confinement are plotted along with the integrated modeling results for HI-SEAS. The theories considered here include Bechtel’s theory of difficult time psychologically during the 3rd quarter. Rohrer’s theory of depression followed by volatility, and the theory of relief from pressure after halfway point. The HI-SEAS data most closely follows a combination of Relief from Pressure theory and Rohrer’s theory of volatility. Evidence for each phase of the HI-SEAS stress model is detailed in Table 4. The four phases of the HI-SEAS stress model presented here are 1) Eustress of initial adaptation, 2) Deprivation due to prolonged isolation and confinement, 3) Disruption of team dynamics, and 4) Asynchronous coping.

**TABLE 4:** Psychosocial and biobehavioral evidence for theoretical model of stress during HI-SEAS missions.

Phase	Timing	Indicators	Tagline
Eustress of initial adaptation	First Quarter	Elevated stress hormone levels decreasing (relaxation); physical activity increasing (fitness routine); perceived stress decreasing (positive outlook)	“We’ve got this!"
Deprivation due to prolonged isolation and confinement	Second Quarter	Decreasing dopamine and serotonin (low social reward), decreasing testosterone (assimilation), decreasing 6-SOM (lack of sun exposure), increasing stress hormones (stressors of isolation and confinement)	“Getting frustrated..."
Disruption of team dynamics	Third Quarter	High oxidative damage (wear and tear), reduced physical activity (injuries and/or low drive for exercise) and increasing RHR (fitness declining); Dopamine and serotonin increase first (due to changes in team dynamics) and then decrease while testosterone increases (interpersonal conflict)	“Managing conflict."
Asynchronous coping	Fourth Quarter	High perceived stress (negative outlook), low dopamine and serotonin (depression and anxiety), low 6-SOM (asynchronous schedules), but low oxidative damage and RHR due to variable testosterone and activity (fitness improving, but not as consistent as intial fitness routine)	“Almost out of here..."

The first phase of “eustress” is the performance enhancing stress of Yerkes-Dodson Law (Yerkes and Dodson, 1908). Stress hormone levels are elevated initially due to the major life changes (e.g. leaving family and loved ones, logistics of moving, media appearances, etc.). However, initially elevated stress levels are soon decreasing as participants relax into their new environment; participants are motivated and perform well in fitness routines, social activities, work tasks, time management, etc. This phase lasts for the first quarter of the mission (i.e. first 3 months for a 1-year mission or first 2 months for 8-month mission). The tagline for this phase of the mission is “We’ve got this!”

The second phase of “deprivation” is the impact of prolonged isolation and confinement. With limited sensory stimulation and social interaction, serotonergic and dopaminergic pathways are not being stimulated to produce serotonin and dopamine. In turn, lower dopamine leads to reduced testosterone, and lower serotonin leads to reduced melatonin, which has impacts on circadian rhythm, mood regulation, and antioxidant activity. This phase lasts until approximately the halfway point of the mission. The tagline for this phase of the mission is “Getting frustrated … ”

The third phase of “disruption” is the rupture point where team dynamics, idiosyncratic policies, social norms, and routines begin to break down. The chronic stress is now causing “wear and tear” physiologically in the form of oxidative stress and inflammation, leading to changes in sleep and activity. Changes in team dynamics may initially give a slight boost of dopamine and serotonin due to the stimulating nature of changes in what has been a static, very limited environment. However, as testosterone increases and interpersonal stress arises, this coupled with lack of exercise adds to the vicious cycle of “wear and tear” and distress. This difficult phase of the mission will likely occur after around 6 months of isolation and confinement (or after halfway point but before the last quarter of 8–12 months missions). The tagline for this phase of the mission is “Managing conflict.”

The final phase of the mission is “asynchronous coping” as participants are starting to set their individual priorities for the remaining time in the mission and start looking forward to what they will do after the mission is over. Due to the chronic stress and tension between participants either socially or just due to competing priorities, participants begin to establish asynchronous schedules, with some crewmembers staying up later or others waking up earlier to have more “alone” time. The social participation and fitness routines do start to recover compared to the last phase, but they are not as consistent as they were initially in the mission. Perceived stress is high and somewhat volatile due to inconsistent routines and low cognitive reserves due to chronic stressors. This final phase is for the last quarter of the mission. The tagline for this phase of the mission is “Almost out of here … ”

## Discussion

Multivariate data normalization, integration, and classification methods were applied to evaluate both biobehavioral and psychosocial stress during long-duration Mars analog missions. These data provide supporting evidence for a combination of several aspects of prior theories, such as “relief from pressure”, “third quarter phenomenon”, and end of mission “period of volatility.”

Long-term stress contributes to deficits in behavioral health and performance, such as mental illness, due to chronically engaging neural networks associated with fear and anxiety. As stress activates the fear-sensing amygdala, negative emotions increase, and simultaneously, an override of the prefrontal cortex occurs, which is the commander of logic and reasoning (McEwen, 2005). Mental acuity declines with stress due to impairment of the prefrontal lobe leading to cognitive dysfunction in the form of reduced functional capacity of memory, learning, logic, and reasoning (Shin et al., 2006; Arnsten, 2009). Stress increases the focus on emotions and reduces the ability to reason and override emotional impulses, which contributes to diminished decision-making capability and can exacerbate mental illness.

Another impact of chronically-elevated stress levels is damage to the hippocampus, which is the center for memory and learning. The role of the hippocampus during a stress response is to record detailed memories of stress events and mark when a stress event begins and ends. The hippocampus marking the end of a stress event is required for relaxation and recovery to begin, but the signaling of the amygdala impacts and delays hippocampal memory consolidation phases (Phelps, 2004). In post-traumatic stress disorder (PTSD), a hallmark of the disorder is the inability of the hippocampus to signal that a stress event has ended. Chronic high stress hormone levels eventually overload and damage the hippocampus. MRI studies of PTSD patients’ brains have shown that the hippocampus becomes hyperactive and also is reduced in volume compared to normal, healthy controls (Gilbertson et al., 2002; McEwen, 2003). The hippocampus has a limited capacity to regenerate and rebuild over time, though this capacity lessens with age (Kino, 2015). Young people have been shown to have a higher regenerative capacity, but a vulnerability window exists in children in which trauma will hinder brain development causing long-term impacts (Danese and McEwen, 2012). People who faced trauma in childhood have been shown to have lower hippocampal volume and lower neural connectivity in the hippocampus as well as lower cognitive performance in adulthood (Aas et al., 2012).

To further develop and evaluate theories on adaptation to isolation and confinement, this research has developed a data integration and modeling approach to identify biobehavioral and psychosocial stress during long-duration Mars analog missions. The “cVar normalization” method presented here enables data integration for multivariate, longitudinal data streams of varying magnitude and distribution. The application of k-means clustering algorithm has enabled the classification of stress indicators by labeling this multi-variate, non-normally distributed, longitudinal dataset.

Stress hormone levels are highest at the beginning and the halfway point of the mission. High levels of cortisol have been shown to impact serotonin re-uptake, essentially metabolizing too quickly and reducing the availability of serotonin (Tafet et al., 2001). Stress responses also lower the stores of dopamine since it is a precursor to the catecholamine noradrenaline. Serotonin and dopamine declined for the first 4 months, then increased at the 5–6 months mark before going back down in 6–9 months timeframe. Testosterone also declined from the beginning of missions until about 4–5 months of elapsed time, then it increased between 4 and 6 months for HM3 and at the 5–7 months point for HM4. Testosterone is lagging dopamine and serotonin in these data, which could indicate that mood-related neurotransmitters impact sex hormone production. For example, the decrease between month 6–7 months in dopamine and serotonin for HM3 was followed by decrease in testosterone from month 7–8. Similarly, an increase in dopamine and serotonin from 4 to 5 months was followed by an increase in testosterone for HM3 from 5 to 6 months. There is some evidence that low testosterone may lead to inactivity (Lightfoot, 2008), that dopamine improves physical activity performance (Heyes, 1985), and that oxidative damage decreases drive for physical activity.

Crewmembers were less physically active at the 6-month point for both HM3 and HM4. Oxidative damage was increasing until the 5-month mark, then it went down for the remainder of mission. Physical activity is lagging oxidative damage in these data, which could lead to the hypothesis that drive for physical activity decreases when oxidative damage is high. For example, oxidative damage increased from month 4–5 for both HM3 and HM4, then physical activity went down from month 5–6. For HM4, Resting Heart Rate (RHR) decreased for the first 4 months then increased with stress indicators at 7-month and 10-month marks due to the preceding periods of inactivity. For both HM3 and HM4, perceived stress increased steadily during the 5–7 months time period of the missions and then remained high for the rest of the missions. This chronically increasing stress then coincides with less sleep at 6–9 months into the mission which is also when dopamine and serotonin started declining again.

**FIGURE 9:**
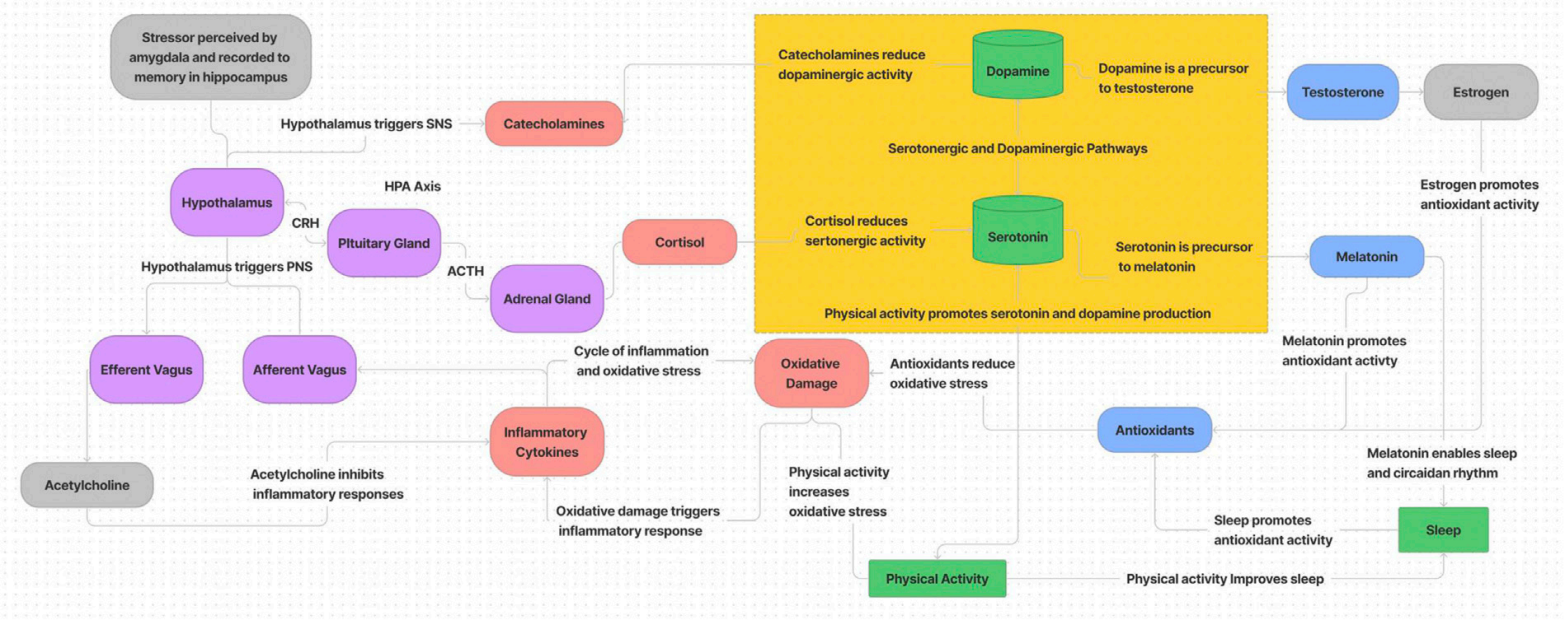
Diagram of biobehavioral and psychosocial stress dynamics.

The dynamics of biobehavioral and psychosocial stress based on the results of longitudinal, multivariate data integration and analysis from HI-SEAS missions is presented in the diagram of Figure 9. While not encompassing all relevant systems and behaviors (i.e. ignoring social, diet/metabolic, and endocannabinoid system influences), this summary is based on the interactions observed from data presented here. In summary, stress responses through the HPA axis trigger catecholamine and cortisol release which reduce dopaminergic and serotonergic activity. This in turn impacts production of melatonin and testosterone (and estrogen), which impacts antioxidant production and circadian rhythm (which reduces antioxidant activity further). While sleep and physical activity initially can promote serotonin and dopamine production to cope with the stress response, eventually oxidative damage accumulates and inhibits drive for physical activity, and without physical activity there is less dopamine and serotonin production, more circadian disruption, more oxidative damage, etc. One of the main processes to counteract this downward spiral is through the parasympathetic nervous system (PNS). The efferent vagal system can stimulate release of acetylcholine to reduce inflammation and stop the cascading cycle of oxidative damage and inflammation (Pavlov et al., 2003; Namgung et al., 2022). If left unchecked, inflammation causes more oxidative damage, which further reduces drive for physical activity and continues the cycle. Some emerging therapies to treat stress-related disorders are leveraging PNS pathways, with electrical or magnetic stimulation and/or pharmacologically administered therapies.

Physiologically, the vagus nerve has two modes: 1) “fawn” which is when ventral vagal system is on overdrive for people pleasing to “tend and befriend”, or 2) “freeze” which is when dorsal vagal system induces “rest and digest” to counteract “fight or flight” responses. In the extreme, overstimulation of the vagus nerve can cause episodes of syncope, including physically fainting, mentally dissociating, and/or secreting analgesics in anticipation of pain. More commonly, this response acts as a “brake system” to pause or “stiffen” the body to momentarily slow down heart rate in order to prepare the body for action (Schwartz, 2021). The “fawn” response involves being overly aware of the social and emotional needs of others, prioritizing others above one’s own needs, and may include a disconnect from physical sensations (Kilrain, 2017). When chronically activated, vagal pathways can lead to feelings of exhaustion and burnout.

Being on a small team in isolation creates intense social conditions where teammates are constantly subjected to each other’s presence and cannot escape for recharge and relaxation. Crews are forced to trial and error different ways of dealing with conflicts or avoiding conflict that lead to criticism, disagreement, defensiveness, stonewalling, etc. The team dynamics of a small group are pressurized while confined together and present challenges that are familiar to households, such as choosing to compartmentalize and avoid conflict vs. put in the work for conflict resolution vs. escalating conflict to a breaking point. Insights and approaches from family therapy may provide strategies to provide relief from the social pressures for these small teams living in isolated and confined environments.

## Conclusion

Both spaceflight and military applications are the extreme of remote healthcare, and therefore stress monitoring technologies developed for these contexts may also be useful for practicing rural medicine or telemedicine. Being able to monitor health and stress remotely may support both preventative self-care and disease management. This work may also be insightful for mental health professionals working to help patients manage post-traumatic stress disorder (PTSD) and other stress/anxiety disorders, as stress monitoring tools may assist in diagnosis and recovery from PTSD or other psychiatric disorders.

Multivariate data normalization, integration, and classification methods were applied to evaluate both biobehavioral and psychosocial stress during long-duration Mars analog missions. These data provide supporting evidence for a combination of several aspects of prior theories, such as “relief from pressure”, “third quarter phenomenon”, and end of mission “period of volatility.” A practical outcome of this research is a set of recommendations for how to monitor health and stress efficiently and reliably on a daily to weekly basis over long periods of time and supports that around the halfway timepoint or 4–6 months is when it is most critical to intervene with stress management strategies. To proactively manage stress in isolation and maintain crew cohesion in confinement, the team will need to have flexible, yet supportive policies (such idiosyncratic policies for code of conduct, including prioritizing and scheduling daily activities) that allow for adjustments in individual, mission, and team dynamics. There’s a need for countermeasures during this difficult time period, and one approach could mimic the role of a “coach” or another force for keeping focus on the mission goals to help promote a positive outlook and lead to efficiently performing as a determined and unified team. Maintaining an integrated perspective, rules and hierarchy may require adapting to support individual and team needs, and working together collectively to achieve a mission goal or other external objective for long spaceflight missions or other high-risk, complex operations.

This research has designed data collection protocols for biological sampling, wearables data, and self-reporting. Each of these data streams have various logistical requirements both for the crew and for post-mission data analysis as well as data quality assurance and privacy concerns. By collecting biological samples for metabolite profiling, wearables data for behavioral monitoring, and survey responses for self-reporting, this research investigation has developed longitudinal, multivariate measures of biobehavioral and psychosocial stress over time.

Some best practices for stress research over long periods of time will be provided here by data category starting with 1) self-reporting, then 3) wearables for activity and 4) for sleep, and finally 5) biosamples. First, using visual analog scales, rather than long-form questionnaires is recommended for longitudinal studies. Due to the repetition over time, when using standard likert scale questionnaires participants will start to answer long-form questionnaires in a routine way, whereas VAS (ideally with slider scales that do not show the number selected) generate more reflective and varied responses over time. Second, resting heart rate measured first of the morning upon awakening is the best indicator of activity levels and overall health and fitness. Thirdly, changes in sleep schedules such as shifting to later bed times or earlier wake times is an indicator of team dynamics and potentially conflict avoidance. Lastly, for biosamples in this context, the key metabolites for tracking include cortisol and cortisone, especially for the first quarter to measure relaxation response, then dopamine, serotonin, 3-HPMA, and 6-SOM throughout the mission. This study used both hair and urine; however, in the future, it is recommended to collect urine only in the future due to the additional metabolites that can be measured in urine compared to hair. Also, hair analysis has inherent errors due to differential hair growth rates. It is assumed that trimming hair from the scalp results in measures that are lagging approximately two weeks behind due to the remaining new hair growth being still inside the follicle; however, this time lag likely varies from person to person based on their hair growth rates.

Going beyond to discover the underlying causes of individual and crew differences in adapting to isolation and confinement at HI-SEAS requires further investigation with performance measures such as team performance tasks and also qualitative data, such as journal entries to triangulate the structure of how biomarkers, behaviors, and other latent factors combine and ladder up to the biobehavioral and psychosocial stress responses of HI-SEAS crews. For structural equation modeling, the latent, unobserved variables include unknown circumstances with habitat maintenance, mission support and crew dynamics, disagreements and conflict among the crew, individual vulnerabilities and attachment styles, coping skills, intrinsic motivation, workload, hygiene, and prior experience (including prior trauma and triggers) with isolation and confinement or other relevant contexts and environments.

## Data Availability

The datasets presented in this article are not readily available because datasets generated and analyzed for this study will be provided to the NASA Life Sciences Data Archive upon completion of this work. Until then, the author will directly accommodate requests to access de-identified data from these studies. Requests to access the datasets should be directed to joce.rosenberg@gmail.com.
